# Prognosis of HIV Patients Receiving Antiretroviral Therapy According to CD4 Counts: A Long-term Follow-up study in Yunnan, China

**DOI:** 10.1038/s41598-017-10105-7

**Published:** 2017-08-29

**Authors:** Li Ren, Juan Li, Shiyi Zhou, Xueshan Xia, Zhenrong Xie, Pan Liu, Yu Xu, Yuan Qian, Huifeng Zhang, Litang Ma, Qiuwei Pan, Kunhua Wang

**Affiliations:** 1 Faculty of Environmental Science and Engineering, Kunming University of Science and Technology, Kunming, 650093 Yunnan Province China; 2 The First People’s Hospital of Yunnan Province, Kunming, 650031 Yunnan Province China; 3 Medical faculty of Kunming University of Science and Technology, Kunming, 650093 Yunnan Province China; 4 Department of Gastroenterology and Hepatology, Erasmus University Medical Center, Rotterdam, The Netherlands; 5 Yunnan Institute of Digestive Disease, the First Affiliated Hospital of Kunming Medical University, Kunming, 650032 Yunnan Province China; 6 Faculty of Life Science and Technology, Center for Molecular Medicine in Yunnan province, Kunming University of Science and Technology, Kunming, 650093 Yunnan Province China; 7 Yan’an Hospital of Kunming Chenggong hospital, Kunming, 650501 Yunnan Province China; 8 The First People’s Hospital of Zhaotong City, Zhaotong, 657000 Yunnan Province China

## Abstract

We aim to evaluate the overall survival and associated risk factors for HIV-infected Chinese patients on antiretroviral therapy (ART). 2517 patients receiving ART between 2006 and 2016 were prospectively enrolled in Yunnan province. Kaplan-Meier analyses and Cox proportional hazard regression analyses were performed. 216/2517 patients died during a median 17.5 (interquartile range [IQR] 6.8–33.2) months of follow-up. 82/216 occurred within 6 months of starting ART. Adjusted hazard ratios were10.69 (95%CI 2.38–48.02, p = 0.002) for old age, 1.94 (95%CI 1.40–2.69, p < 0.0001) for advanced WHO stage, and 0.42 (95%CI 0.27–0.63, p < 0.0001) for heterosexual transmission compared to injecting drug users. Surprisingly, adjusted hazard ratios comparing low CD4 counts group (<50 cells/µl) with high CD4 counts group (≥500 cells/µl) within six months after starting ART was 20.17 (95%CI 4.62–87.95, p < 0.0001) and it declined to 3.57 (95%CI 1.10–11.58, p = 0.034) afterwards. Age, WHO stage, transmission route are significantly independent risk factors for ART treated HIV patients. Importantly, baseline CD4 counts is strongly inversely associated with survival in the first six months; whereas it becomes a weak prognostic factor after six months of starting ART.

## Introduction

As estimated by the Chinese health authority^1^, there were 501,000 people living with human immunodeficiency virus (HIV/AIDS) by the end of 2014 in China, accounting for 0.037% of the total population. Despite of a low national prevalence rate, the HIV epidemic is severe in some areas of the Southwest China, in particular the Yunnan, Sichuan, and Guangxi provinces. Yunnan is the area most affected by HIV/AIDS in China. The epidemic has spread from high-risk groups including drug users, sex workers and unsafe blood recipients to the general population. With a population of 44 million, officials estimate that this province has 80,000 HIV infected individuals. Antiretroviral drugs in combination of three or more drugs from more than one class, often called “highly active antiretroviral therapy (HAART)”, are very effective in suppressing HIV, although do not eradicate the virus. Since the first introducing of HAART in the mid-1990s, it has led to an unprecedented decline of mortality caused by HIV/AIDS both in the USA^2^ and Europe^3^. Because of the great success of HAART in treating HIV/AIDS in the developed countries, the World Health Organization (WHO) has promoted a public health approach to scale up the access of antiretroviral therapy in resource-limited setting since 2002^4, 5^. As an emergency response to save and improve the lives of AIDS patients in China, the China National Free Antiretroviral Treatment Program (NFATP) was piloted in 2002 and scaled up in 2003 national wide^6, 7^. Until 2014, a total of 295,358 patients in China have received HAART^1^, and thus a large proportion of HIV patients have benefited from this program. Based on China national HIV database as well as a few local studies, an increasing coverage of antiretroviral treatment has significantly decreased HIV/AIDS-related mortality^8–12^.

It is important to identify prognostic factors for survival among HIV-infected patients receiving ART. Based on previous studies, the main risk factors for death include baseline low CD4 cell count, old age and advanced WHO stage. Among them, CD4 cell count was suggested as the most important prognostic factor based on the estimated hazard ratio values^9–11, 13–21^. According to those results, the prognostic value of CD4 cell count seems to be well-established. However, a recent study, examining European and North American patients, suggested that patients with low baseline CD4 cell count only carry the burden of increased risk of death up to 5 years after ART^16^. This indicated the impersistence of the CD4 cell count on the increased mortality, although no other study has reported similar observation. In order to better understand the treatment outcome of HIV patients, we have carried out a large prospective cohort study with long-term follow up in China, enrolling patients from Zhaotong, a prefecture-level city located in the northeast corner of Yunnan province. In this study, we aim to evaluate the overall survival and associated risk factors for the HIV-infected patients on ART in this cohort, with particular focus on the prognostic value of CD4 cell count.

## Results

### Baseline Characteristics

A total of 2517 patients (adult) before starting ART were enrolled between July 2006 and April 2016 in this study. The baseline characteristics of the study population at start of ART are summarized in Table 1. Patients had a median age of 39 (interquartile range 31–50) years. The number of male patients accounted for 59.9% of all patients. A total of 76.2% patients were married. As for the HIV transmission routes, more than two-thirds (71.9%) were infected through heterosexual; whereas 10.5% through injecting drug use (IDU). In terms of WHO clinical stage, 78.9% received ART at stage I/II and 20.9% at stage III/IV. When measuring CD4 cell count on continuous scale, CD4 cell count had an overall median 281 (IQR 177-388) per µl. By dividing CD4 cell count into subgroups, there are 5% of patients presenting low CD4 cell count (<50 per µl), and 66.9% presenting high CD4 cell count (≥200 per µl).

**Table 1:** Baseline characteristics of the patients according to whether lost to follow up or not.

Characteristics	Overall (n = 2517)	LTFU (n = 199)	Non-LTFU (n = 2318)	p-value^d^
Gender, no. (%)				0.512^a^
Male	1507/2517 (60)	124/199 (62)	1383/2318 (60)	
Female	1010/2517 (40)	75/199 (38)	935/2318 (40)	
Marital status, no. (%)				0.004^a^
Unmarried	296/2501 (12)	39/197 (20)	257/2304 (11)	
Married	1919/2501 (76)	137/197 (70)	1782/2304 (77)	
Divorced	156/2501 (6)	10/197 (5)	146/2304 (6)	
Widowed	130/2501 (5)	11/197 (6)	119/2304 (5)	
Age at ART initiation, year				0.137^b^
Mean (SD)	42 (14)	40 (15)	42 (14)	
Median (IQR)	39 (31–50)	36 (29–49)	39 (31–50)	
WHO HIV Clinical Stage, no. (%)				0.004^a^
Stage I/II	1986/2511 (79)	158/197 (80)	1828/2314 (80)	
Stage III/IV	525/2511 (21)	39/197 (20)	486/2314 (21)	
CD4, cells/µl				0.010^a^
Median (IQR)	281(177–388)	330 (220–455)	278 (173–383)	
<50	126/2402 (5)	5/179 (3)	121/2223 (1)	
50–199	593/2402 (24)	31/179 (17)	562/2223 (25)	
200–349	895/2402 (37)	67/179 (37)	828/2223 (37)	
350–499	478/2402 (20)	44/179 (25)	434/2223(20)	
≥500	310/2402 (13)	32/179 (18)	278/2223 (13)	
Transmission category, no. (%)				
Injecting drug users (IDU)	263/2517 (11)	42/199 (21)	221/2318 (10)	<0.0001^a^
Homosexual	54/2517 (2)	3/199 (2)	51/2318 (2)	
Heterosexual	1809/2517 (72)	136/199 (68)	1673/2318 (72)	
Others/unknown^c^	391/2517 (16)	18/199 (9)	373/2318 (16)	

Abbreviations: LTFU = lost to follow up; Non-LTFU = not lost to follow up; ART indicates antiretroviral treatment; SD = standard deviation; IQR = interquartile range. ^a^Indicates Pearson’s Chi-squared test; ^b^Indicates Welch Two Sample t-test; ^c^Include blood transfusion, mother-to-Child, and others; ^d^Baseline characteristics were compared between patients of LTFU and patients without LTFU.

### Cumulative mortality in study population

**Figure 1:**
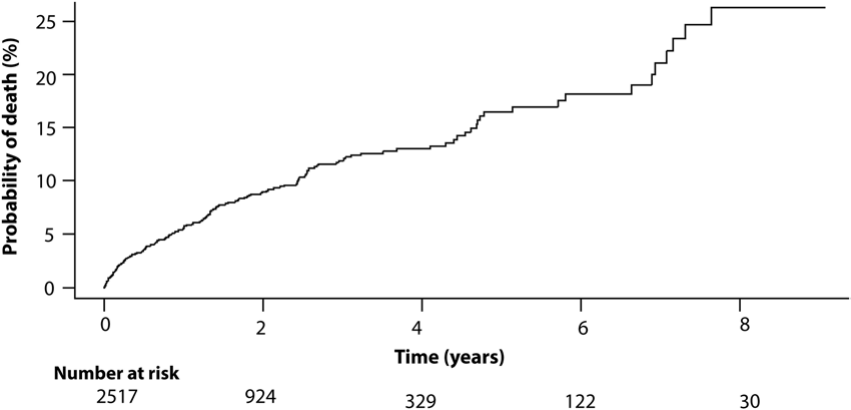
Cumulative morality from all-cause mortality for study population with human immunodeficiency virus (HIV) infection receiving antiretroviral therapy (ART). Corresponding numbers at risk at different time-points have been indicated below the graph.

The crude cumulative mortality for study population is displayed in Fig. 1. Their median follow-up time was 17.5 (IQR 6.8–33.2) months. The cumulative probabilities for mortality were 9%, 13%, 19%, and 26% at 2 years, 4 years, 6 years, and 8 years, respectively. 8.6% (216/2517) of patients died during the follow-up period. Within these patients, 38.0% (82/216) died in their first 6 months. Patients who died at their first six months of follow-up period had a median age of 42.0 (IQR 34.3–60.0), 70.7% were male, and 57.3% were in advanced WHO clinical stage of III/IV. The continuous CD4 cell count had a baseline median 140 (IQR 59–236) cells/µl. 25.6% (21/82) patients were in low CD4 cell count stratum (lower than 50 per µl); while 26.8% (22/82) in high CD4 cell count stratum (200/µl or greater) (supplementary Table S1).

### Prognostic values of baseline factors

**Figure 2:**
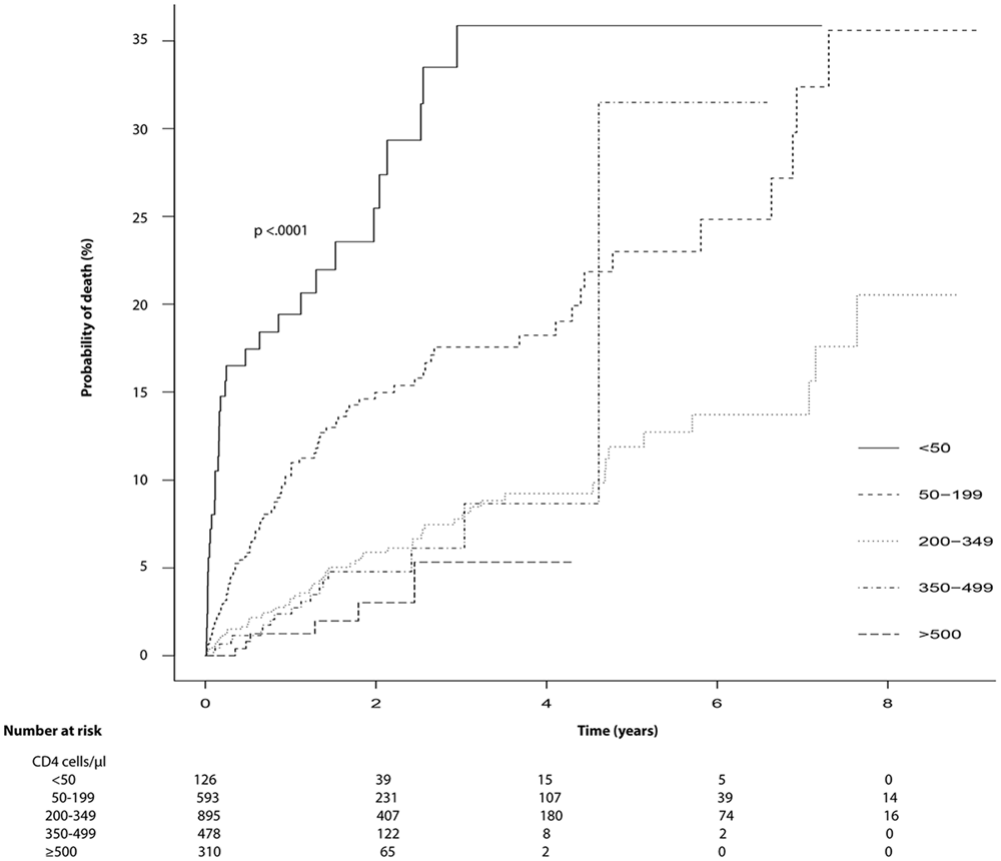
Cumulative morality from all-cause mortality for study population infected by human immunodeficiency virus (HIV) receiving antiretroviral therapy (ART) according to CD4 cell count (*p* < 0.0001). Corresponding numbers at risk at different time-points split by CD4 cell count have been indicated below the graph.

The associations of baseline factors at start of ART with mortality, estimated from crude and adjusted Cox models with time-dependent coefficients for CD4 cell count are shown in Table 2. Age and CD4 cell count were significant prognostic factors for survival (p < 0.0001 by log-rank test) based on crude survival analysis as shown in Fig. 2 and Supplementary Figure S1. Of note, patients with low CD4 cell count (<50 cells/µl) had drastically increased risk for mortality at early follow-up time. In multivariate model, advanced WHO clinical stage (HR 1.94, 95%CI 1.40–2.69, p < 0.0001) and old age (>75 years) (HR 10.69, 95%CI 2.38–48.02, p = 0.002) were significantly associated with worse survival; heterosexual mode was associated with better survival (HR 0.42, 95%CI 0.27–0.63, p < 0.0001) than injecting drug users. Varying hazard ratios for baseline CD4 cell count were observed in analysis for different time intervals since the start of ART. For instance, the adjusted HRs comparing low CD4 cell count group (<50 cells/µl) with high CD4 cell count group (≥500 cells/µl) during the first six months was 20.17 (95%CI 4.62–87.95, p < 0.0001), but it became 3.57 (95%CI 1.10–11.58, p = 0.034) after the first six months. By comparing those with CD4 cell count (50–199 cells/µl) and high CD4 cell count (≥500 cells/µl), significant HRs were found in the two intervals: 5.06 (95%CI 1.20–21.32, p = 0.027) ≤ 0.5 year and 2.89 (95%CI 1.02–8.04, p = 0.045) > 0.5 year (Table 2). With respect to the model accuracy, concordance with 0.791 suggested good predictive performance of the Cox model.

**Table 2:** Cox proportional hazard regression analyses analyzing all-cause mortality after starting ART.

Baseline characteristics	Univariate analysis		Multivariate analysis	
	Hazard ratio (95% CI)	p value	Hazard ratio (95% CI)	p value
Gender				
Female	1	(Reference)	1	(Reference)
Male	1.74 (1.30–2.34)	<0.001	1.26 (0.90–1.75)	0.167
Marital status				
Married	1	(Reference)	1	(Reference)
Unmarried	1.14 (0.78–1.68)	0.506	—	—
Divorced	1.11 (0.64–1.92)	0.706	—	—
Widowed	1.81 (1.05–3.14)	0.033	—	—
Age at ART initiation, year^a^				
(0,20]	1	(Reference)	1	(Reference)
(20,25]	1.07 (0.23–4.97)	0.929	0.96 (0.20–4.47)	0.961
(25,30]	1.47 (0.35–6.21)	0.604	0.83 (0.20–3.63)	0.818
(30,35]	1.99 (0.48–8.28)	0.344	0.88 (0.21–3.79)	0.872
(35,40]	2.10 (0.50–8.78)	.308	1.05 (0.25–4.47)	0.948
(40,45]	2.26 (0.53–9.59)	.271	1.18 (0.27–5.09)	0.828
(45,50]	1.81 (0.41–8.11)	0.435	0.77 (0.16–3.60)	0.739
(50,55]	2.23 (0.49–10.17)	0.302	1.45 (0.31–6.66)	0.636
(55,60]	4.40 (1.02–18.91)	0.046	2.92 (0.67–12.66)	0.153
(60,65]	3.71 (0.82–16.57)	0.087	1.84 (0.40–8.51)	0.436
(65,70]	5.42 (1.20–24.49)	0.028	2.96 (0.64–13.70)	0.165
(70,75]	6.73 (1.49–30.40)	0.013	2.45 (0.51–11.74)	0.264
(75,90]	16.25 (3.73–70.85)	0.000	10.69 (2.38–48.02)	0.002
WHO HIV Clinical Stage				
Stage I/II	1	(Reference)	1	(Reference)
Stage III/IV	3.15 (2.40–4.13)	<0.0001	1.92 (1.38–2.66)	<0.0001
Transmission				
Injecting drug users (IDU)	1	(Reference)	1	(Reference)
Homosexual	0.34 (0.08–1.41)	0.137	0.38 (0.09–1.59)	0.184
Heterosexual	0.60 (0.43–0.84)	0.004	0.42 (0.28–0.64)	<0.0001
Others/unknown^b^	1.06 (0.71–1.59)	0.771	0.62 (0.38–1.02)	0.060
Baseline CD4, cells/µl				
≤0.5 years				
≥500	1	(Reference)	1	(Reference)
350–499	1.61 (0.31–8.30)	0.569	1.40 (0.27–7.25)	0.685
200–349	2.53 (0.58–11.07)	0.217	2.14 (0.49–9.39)	0.312
50–199	8.41 (2.01–35.07)	0.003	5.06 (1.20–21.32)	0.027
<50	28.51 (6.68–121.60)	<0.0001	20.17 (4.62–87.95)	<0.0001
>0.5 years				
≥500	1	(Reference)	1	(Reference)
350–499	1.89 (0.62–5.79)	0.267	1.73 (0.56–5.32)	0.338
200–349	1.91 (0.70–5.35)	0.220	1.71 (0.61–4.81)	0.310
50–199	3.93 (1.41–10.94)	0.009	2.89 (1.02–8.04)	0.045
<50	4.94 (1.57–15.57)	0.006	3.57 (1.10–11.58)	0.034

Abbreviations: 95% CI = 95% confidence interval; ART = antiretroviral therapy.

^a^The patient number in each age subgroup were the following: (0,20] 50; (20,25] 185; (25,30] 369; (30,35] 383; (35,40] 389; (40,45] 295; (45,50] 226; (50,55] 158; (55,60] 147; (60,65] 122; (65,70] 80; (70,75] 67; and (75,90] 45.

^b^Includes blood transfusion, mother-to-Child, and others/unknown.

## Discussion

This is a large prospective study pioneering the assessment of patient survival and associated risk factors in Chinese population with HIV infection receiving ART. We described the treatment outcome and identified baseline age, WHO clinical stage as independent predictors for patients survival for all time. The low CD4 cell count at baseline had a strong inverse association with survival at first six months of starting ART.

The survival benefit from ART have been demonstrated intensively among the HIV infected patients^2, 3^. A high tolerance of ART regimen and fewer regimen switches were found among our patients (Supplementary Figure S2). Of the patients who died during the follow-up, more than two thirds occurred within the first six months since ART initiation, similar to previous studies^11, 14, 22^. The patient characteristics between those dead at first six months and the entire study population were clearly described in this study. The former group had a higher percentage of patients with old age, advanced WHO clinical stage (stage III/IV) and low CD4 cell count compared with the entire study population. Thus, poor baseline patient characteristics seem to contribute largely to the worse survival, supporting the importance of early diagnosis and treatment^11, 14, 15, 23–25^. Overall, we feel that the first six months of ART is critical for improving survival outcome in HIV-infected patients.

The varying coefficients for CD4 cell count was demonstrated among our patients. Many previous studies only reported CD4 cell count as a strong prognostic factor for all time of their study period, but the potential varying effects of CD4 cell count on survival were hardly discussed in their studies^9, 11, 21, 26, 27^. A potential reason could be the lack of testing the proportional hazard assumption for Cox model, or such assumption was met in those studies. Another potential explanation could be the relatively short follow-up time in many previous studies. Taking this into consideration, a recent retrospective study with long-term follow-up (more than ten years) from the Antiretroviral Therapy Cohort Collaboration (ART-CC) have suggested that the baseline CD4 cell count is less prognostic after five years since starting ART^16^. In other words, the patients with low baseline CD4 cell count, who survived the first five years since ART, may expect similar mortality to that of patients with high baseline CD4 cell count. In our prospective cohort study, a strong inverse association between baseline CD4 cell count and risk for mortality was also observed, but only for the first half year after starting ART. We observed a 10-fold increased mortality for low CD4 cell count, which was much higher than the 2.8-fold increase in the ART-CC study^16^. Of note, there are several differences between these two studies, in particular European Americans vs Chinese population. Another important difference to be noticed is the anti-HIV drugs used in those studies. The patients engaged in the ART-CC study received ART between 1996 and 2001; however, our patients have been treated since 2006 with the newer anti-HIV drugs. Given the fact that new drugs introduced since 2002 provide a better immunological response^28, 29^, our data might reflect more the treatment effect on HIV infected patients nowadays. These factors may contribute to the discrepancies observed between our study and the previous ones. Besides, the CD4 cell count at six months after starting ART was also examined in our study for assessing its potential association with survival after six months of starting ART. In contrast to values at baseline, the six-month CD4 cell count was strongly associated with the worse survival for the period after six months of ART initiation. Consistently, the prognostic value of six-month CD4 cell count has been demonstrated previously^30^. To be noticed, due to the missing values of six-month CD4 cell count among the patients who had survived for six months (displayed in Supplementary Figure S3), these data were not shown in this study.

A potential limitation of this study is the lacking of baseline viral load. Although the primary aim of ART is to inhibit the viral replication and reduce viral load, the resulted increase of CD4 cell count is the main goal as it serves as the most important indicator of immune function in HIV-infected patients. Despite this, WHO clinical stage and particularly CD4 cell counts were analyzed. Therefore, the potential bias from not analyzing viral load have been largely circumvented by including the analysis of WHO clinical stage and particularly CD4 cell counts. Besides, the present study only analyzed the data which were collected at start of ART initiation. Although baseline CD4 cell count has been proven to be an important predictor for the long-term outcome of ART and patient survival^19, 31, 32^, the dynamics of CD4 cell count across follow-up period could also be very important, deserving further investigation.

In conclusion, this is a large prospective study with long-term follow-up investigating the treatment outcome and prognostic factors for the HIV-infected patients receiving ART. Baseline characteristics including gender, age and WHO clinical stage are significantly associated with all-cause mortality. Importantly, we reported the time-dependent coefficients for CD4 cell count over different time intervals among Chinese population. The strong inverse association between CD4 cell count and risk for mortality has been demonstrated for the first half year after starting ART. However, CD4 cell count at six month has a strong inverse association with survival after six months of starting ART.

## Methods

### Study population

A prospective cohort study was conducted by enrolling HIV patients at start of ART between 2006 July to 2016 April in Zhaotong, Yunnan province, China. Patients were enrolled from different areas of Zhaotong (a prefecture-level city), including Zhaoyang District, Ludian County, Qiaojia County, Yanjin County, Daguan County, Yongshan County, Suijiang County, Zhenxiong County, Yiliang County, Weixin County and Shuifu County. All patients were treated based on the criteria of the “National AIDS Free Antiviral Treatment Manual”, HIV drug resistance, individual health, and other factors. We collected data on demographics (age, sex, marital status, transmission category), histological parameter (WHO clinical stage), and laboratory maker (CD4 cell count) at baseline when patients initiated ART.

The institutional ethical committee of the First Affiliated Hospital of Kunming Medical University has approved this study. Informed consent was obtained from all participants in this study. No personally identifiable information was seen and used in our data analysis. All the methods of this study were performed in accordance with the guideline and regulation of the institutional ethical committee of the First Affiliated Hospital of Kunming Medical University.

### Statistical Analysis

All patients were followed up from the date since starting ART until the date of death, loss to follow-up, or the end of follow-up. Patients who were lost to follow-up or the event did not occur within the study duration were considered as censored cases. For the patients with treatment changes or interruptions, we analyzed the data as their intent to continue treatment, same as the other patients.

In descriptive statistics, continuous variables were reported as mean with standardized deviation (SD) and median with interquartile range (IQR). Categorical variables were reported as number with percentage. A crude survival analysis (Kaplan-Meier curve) was utilized to analyze the patients’ survival on ART during 10-year follow up. An adjusted survival analysis, Cox proportional hazard regression analysis, was used to evaluate the factors related to survival outcome. Variables with a p-value below 0.20 on univariate analysis were included in multivariate analysis^33–35^. All statistical analyses were performed in R (version 3.3.1)^36^. Particularly, “survival” package^37^ was used for survival analyses. The loss to follow up and not loss to follow up against survival time were plotted to depict the their patterns (Supplementary Figure S4). The assumption of proportional hazards was tested both statistically (Supplementary Table S2) and graphically (Supplementary Figure S5) using function *cox*.*zph*. Besides, Concordance was calculated to assess the Cox model accuracy. One of the strengths of Cox model is its ability to encompass the time-varying coefficients. A *step function* was used to analyze the time-dependent effect of baseline CD4 cell count over different time intervals after breaking the data set into time dependent parts using *survSplit* function^38^. *P* < 0.05 (two-tailed sides) was considered as significant. The R code for performing Cox proportional regression analyses in this study is shown in Supplementary Figure S6.

### Data availability

The datasets generated during and/or analyzed during the current study are available from the corresponding author on reasonable request.

### Role of the Funder/Sponsor

The funding organizations had no role in the design and conduct of the study; collection, management, analysis, and interpretation of the data; preparation, review, or approval of the manuscript; and decision to submit the manuscript for publication.

## Electronic supplementary material

Supplementary info
