# Supplementary material for: Prognosis of HIV Patients Receiving Antiretroviral Therapy According to CD4 Counts: A Long-term Follow-up study in Yunnan, China
Source: Sci Rep. 2017 Aug 29;7:9595. doi: 10.1038/s41598-017-10105-7 (PMC5575268; doi:10.1038/s41598-017-10105-7)
Supplement: Supplementary file 1 — Supplementary info [file 41598_2017_10105_MOESM1_ESM.pdf]

Prognosis of HIV Patients Receiving Antiretroviral Therapy According to CD4 Counts: A Long-term Follow-up study in Yunnan, China

Li Ren[1,2,3#], Juan Li[4#], Shiyi Zhou[5], Xueshan Xia[6], Zhenrong Xie[5], Pan Liu[7], Yu Xu[5], Yuan Qian[8], Huifeng Zhang[2], , Litang Ma[8], Qiuwei Pan[4], Kunhua Wang[1,5*]

[1]Faculty of Environmental Science and Engineering, Kunming University of Science and Technology, Kunming, 650093, Yunnan Province, China;

[2]The First People's Hospital of Yunnan Province, Kunming, 650031, Yunnan Province, China;

[3]Medical faculty of Kunming University of Science and Technology, Kunming, 650093, Yunnan Province, China;

[4]Department of Gastroenterology and Hepatology, Erasmus University Medical Center Rotterdam, The Netherlands;

[5]Yunnan Institute of Digestive Disease, the First Affiliated Hospital of Kunming Medical University, Kunming, 650032, Yunnan Province, China;

[6]Faculty of Life Science and Technology, Center for Molecular Medicine in Yunnan province, Kunming University of Science and Technology, Kunming, 650093, Yunnan Province, China;

[7]Yan'an Hospital of Kunming Chenggong hospital, Kunming, 650501, Yunnan Province, China;

[8]The First People's Hospital of Zhaotong City, Zhaotong, 657000, Yunnan Province, China.

[#]These authors contributed equally.

***Correspondence:**

Kunhua Wang, Yunnan Institute of Digestive Disease, the First Affiliated Hospital of Kunming Medical University, Kunming, 650032, Xichang Road 295, Yunnan Province, China.

Tel: (+86)871-6532 4888 Email: kunhuawangProf@163.com

Running title: Survival of antiretroviral treated HIV Patients

Supplementary materials

Table S1. Baseline Characteristics of the Patients who Died at First 6 Months of Follow-Up Period

**Characteristics**	**Descriptive statistics**
Total	
no.	82
Gender, no. (%)	
Male	58 (70.7)
Female	24 (29.3)
Marital status, no. (%)	
Unmarried	11 (13.4)
Married	61 (74.4)
Divorced	3 (3.7)
Widowed	7 (8.5)
Age at ART initiation, year	
Mean (SD)	47.6 (15.8)
Median (IQR)	42.0 (34.3-60.0)
WHO HIV Clinical Stage, no. (%)	
Stage I/II	35 (42.7)
Stage III/IV	47 (57.3)
CD4, cells/µl	
Mean (SD)	152.7 (128.9)
Median (IQR)	129.0 (44.5-215.0)
<50	21 (25.6)
50-199	32 (39.0)
≥200	22 (26.8)
Transmission category, no. (%)	
Injecting drug users (IDU)	12 (14.6)
Homosexual	0 (0)
Heterosexual	53 (64.6)
Others/unknown	17 (20.7)

ART indicates antiretroviral treatment

Table S2. Testing the Proportional Hazards Assumption of a Cox Regression Model Fit[a]

	rho	chisq	p
Gender			
Female	-	-	(Reference)
Male	-0.014	0.040	.841
Marital status			
Married	-	-	(Reference)
Unmarried	-0.071	1.073	.300
Divorced	0.107	2.25	.134
Widowed	0.066	0.991	.320
Age at ART initiation, year			
WHO HIV Clinical Stage			
Stage I/II	-	-	(Reference)
Stage III/IV	-0.081	1.536	.215
CD4, cells/µl			
≥200	-	-	(Reference)
50-199	-0.104	2.425	.119
<50	-0.150	5.029	.025
Transmission category			
Blood transfusion	-	-	(Reference)
Injecting drug users (IDU)	-0.031	0.192	.661
Homosexual	0.003	0.002	.957
Heterosexual	-0.039	0.296	.586
Mother-to-Child	-0.013	0.036	.850
Others/unknown	-0.022	0.093	.760
GLOBAL[b]	NA	19.958	.096

[a]Compuated by cox.zph() function in package of "survival" from R

[b]Indicates the global test

rho = Spearman's rank correlation coefficient

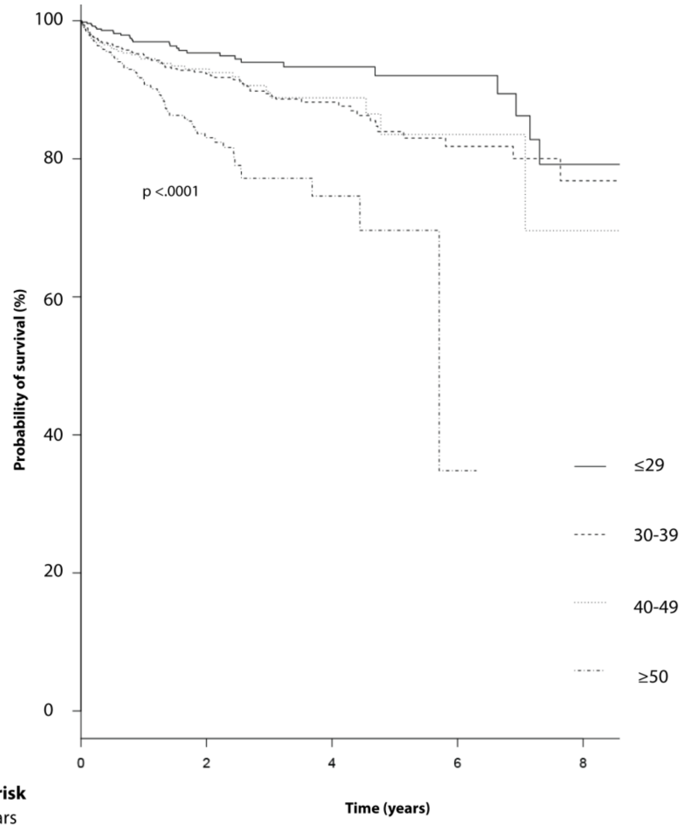

Number at risk

Age, years					
≤29	527	250	93	43	10
30-39	770	355	155	66	16
40-49	551	179	61	12	4
≥50	668	140	20	1	0

Figure S1. Cumulative morality from all-cause mortality for study population infected by human immunodeficiency virus (HIV) receiving antiretroviral therapy (ART) according to age (*p* <.0001). Corresponding numbers at risk at different time-points split by age categories have been indicated below the graph.

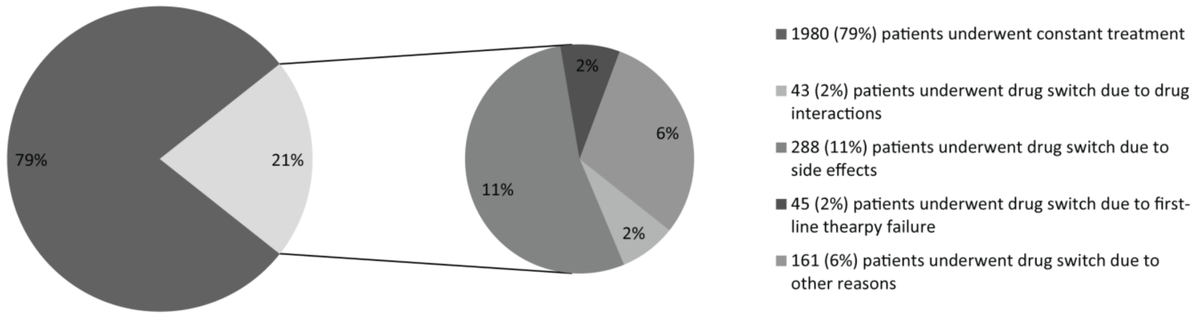

Figure S2. The Pie Chart showing the percentage of patients who switched drugs during Antiretroviral Therapy.

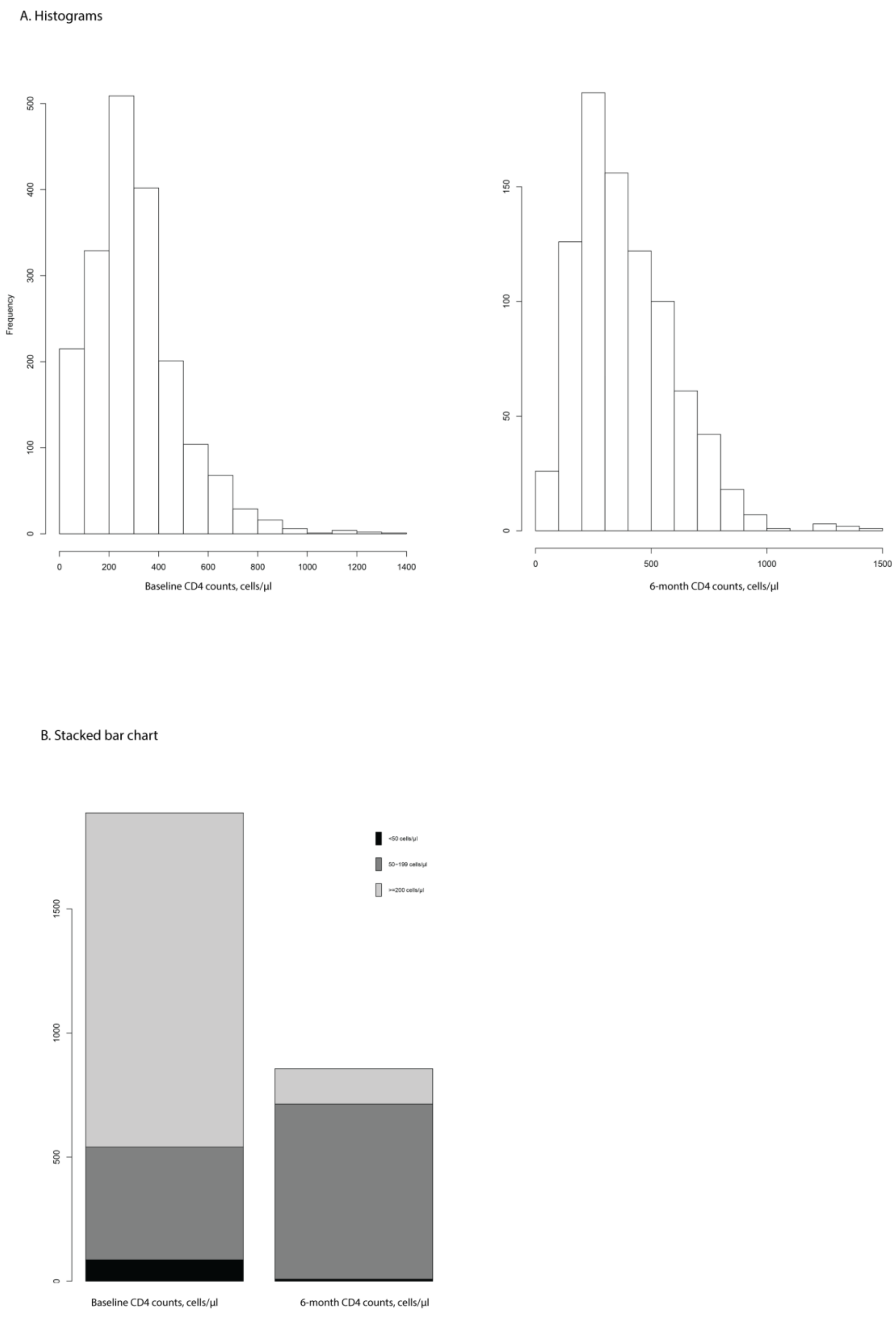

Figure S3: Plots presenting the values of baseline CD4 counts and 6-month CD4 counts for the patients who had survived 6 months after antiretroviral therapy. (A): histograms for continuous data of CD4 counts; (B): stacked bar chart for categorical data of CD4 counts: <50, 50-199, and ≥200 (unit: cells/μl).

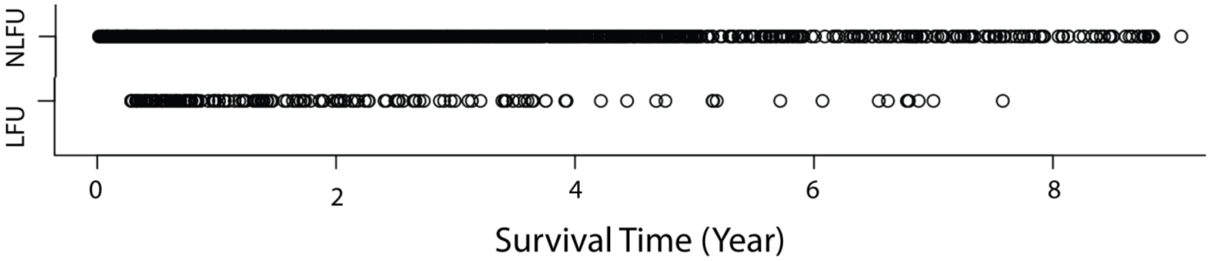

Figure S4: Plot of loss to follow up or not loss to follow up against survival time. LFU = loss to follow up; NLFU = not loss to follow up.

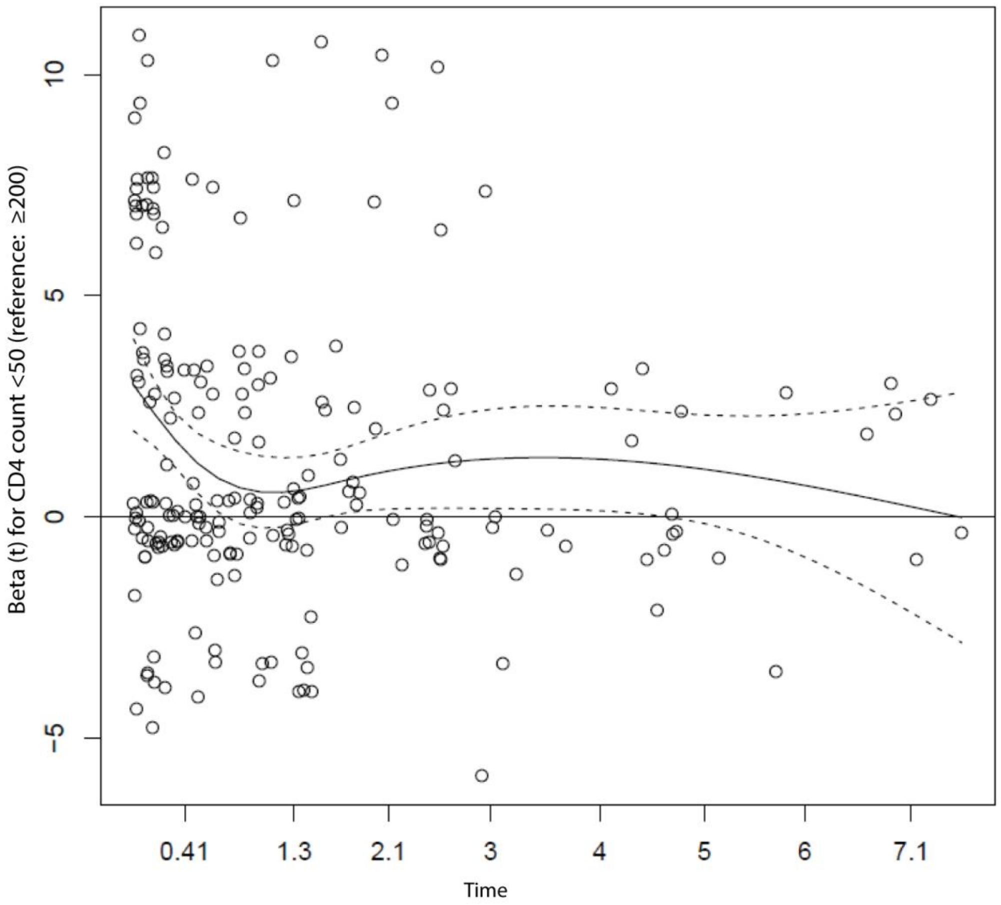

Figure S5: Plot of scaled Schoenfeld residuals against transformed time for covariate CD4 count in Cox model fit. The upper solid line is a smoothing spline fit to the plot, with the broken lines representing a ± 2-standard-error band around the fit; and the bottom solid line is a horizontal line.

```
# Import data..............................................................................................................

data1 <- read.csv ('HIV_data.csv')

# Split dataset into two time intervals: <=0.5 year; >0.5 year................................................

data2<-survival::survSplit(Surv(time,event)~.,data1,cut=c(0.5),

                episode = "tgroup",

                id="id")

# Cox model...............................................................................................................

# 1.Univariate analysis

## CD4 for two time intervals

summmry(coxph(Surv(tstart,time,event)~CD4:strata(tgroup),data=data2)

## Other factors

variables<-c('sex','marriage','transmission','WHO_stage','age')

lapply (variables, function(x)summary(coxph(Surv(time,event)~data2[,x],data=data2)))

# 2.Multivariate analysis

summary(coxph(Surv(tstart,Time_m,Event)~CD4:strata(tgroup)+sex+marriage+transmission+WHO_stage+age, data=data2))
```

Figure S6. R code for performing Cox proportional regression analyses
